# Primary antiphospholipid syndrome in a hemodialysis patient with recurrent thrombosis of arteriovenous fistulas

**DOI:** 10.1590/2175-8239-JBN-2019-0081

**Published:** 2019-07-29

**Authors:** Nikola Gjorgjievski, Pavlina Dzekova-Vidimliski

**Affiliations:** 1 University Hospital of Nephrology, Skopje, Macedonia.

**Keywords:** Antiphospholipid Syndrome, Thrombophilia, Renal Dialysis, Arteriovenous Fistula, Thrombosis, Pre-Eclampsia, Anticoagulants, Síndrome Antifosfolipídica, Trombofilia, Diálise Renal, Fístula Arteriovenosa, Trombose, Pré-Eclâmpsia, Anticoagulantes

## Abstract

**Introduction:**

The antiphospholipid syndrome is a systemic autoimmune disease defined by recurrent vascular and/or obstetrical morbidity that occurs in patients with persistent antiphospholipid antibodies.

**Case presentation:**

A patient on hemodialysis with a primary antiphospholipid syndrome presented with recurrent vascular access thrombosis, obstetrical complications, and positive lupus anticoagulant. The patient had multiple arteriovenous fistulas that failed due to thrombosis. The obstetrical morbidity was defined by one miscarriage at the 7^th^ week of gestation and a pregnancy complicated by pre-eclampsia with preterm delivery at the 28th week of gestation. A thorough thrombophilia screening confirmed the presence of antiphospholipid antibody. Lupus anticoagulant was present in plasma, measured on two occasions 12 weeks apart.

**Conclusion:**

Thrombophilias are inherited or acquired predispositions to vascular thrombosis and have been associated with thrombosis of the arteriovenous fistula. Patients on hemodialysis with recurrent vascular access thrombosis and presence of thrombophilia should be evaluated about the need for anticoagulant therapy with a vitamin K antagonist.

## RESUMO

**Introdução:**

A síndrome antifosfolipídica é uma doença autoimune sistêmica definida por morbidade vascular e/ou obstétrica, recorrente, que acomete pacientes com anticorpos antifosfolípides persistentes.

**Apresentação do caso:**

Uma paciente em hemodiálise com síndrome antifosfolípide primária apresentou trombose recorrente do acesso vascular, complicações obstétricas e anticoagulante lúpico positivo. A paciente apresentava múltiplas fístulas arteriovenosas que falharam devido à trombose. A morbidade obstétrica foi definida por um aborto espontâneo na 7ª semana de gestação e uma gravidez complicada por pré-eclâmpsia com parto prematuro na 28ª semana de gestação. Um rastreamento completo de trombofilia confirmou a presença de anticorpo antifosfolípide. O anticoagulante lúpico estava presente no plasma, medido em duas ocasiões, com 12 semanas de intervalo.

**Conclusão:**

As trombofilias são predisposições hereditárias ou adquiridas para trombose vascular e têm sido associadas à trombose da fístula arteriovenosa. Pacientes em hemodiálise com trombose recorrente de acesso vascular e presença de trombofilia devem ser avaliados quanto à necessidade de terapia anticoagulante com um antagonista da vitamina K.

## INTRODUCTION

The antiphospholipid syndrome (APS) is a systemic autoimmune disease defined by recurrent vascular and/or obstetrical morbidity that occurs in patients with persistent antiphospholipid antibodies (aPLs). aPLs are a heterogeneous group of antibodies against phospholipid-binding proteins on cell membrane, including lupus anticoagulant (LA), anticardiolipin (aCL), and anti-β2-glycoprotein I (anti-β2GPI)^1^.

There are several clinical manifestations of the antiphospholipid syndrome. Thrombotic antiphospholipid syndrome is characterized by venous, arterial, or microvascular thrombosis. Catastrophic antiphospholipid syndrome is presented as thrombosis involving multiple organs. Obstetrical antiphospholipid syndrome is characterized by fetal loss after the 10th week of gestation, recurrent early miscarriages, intrauterine growth restriction, or severe preeclampsia^2^. This syndrome can be primary or associated with other systemic autoimmune diseases, especially with systemic lupus erythematosus (SLE). Between 20 and 30% of patients with SLE have persistent aPLs that are associated with increased morbidity from thrombosis and pregnancy complications^3^.

aPLs can persist in individuals without autoimmune disease and not all individuals with aPL develop thrombotic complications during their lifetime. Among patients without autoimmune disease, the prevalence of aPL positivity is 6% among women with pregnancy complications, 10% among patients with venous thrombosis, 11% among patients with myocardial infarction, and 13% among patients with stroke who are younger than 50 years of age^4^.

The diagnosis of the antiphospholipid syndrome requires persistent antiphospholipid-antibody profiles and any antiphospholipid antibody-related finding. The APS should be included in the differential diagnosis if a patient presents with thrombosis at a young age, an unusual site of thrombosis, recurrent thrombosis, recurrent early miscarriages, late pregnancy loss, and early or severe preeclampsia. Moreover, the following clinical findings, when combined with thrombosis and/or obstetrical complications, may be a clue that a patient has APS: livedo reticularis, heart valve disease, nephropathy, neurological manifestations, signs or symptoms of another systemic autoimmune disease, unexplained prolongation of the activated partial thromboplastin time, or mild thrombocytopenia. The original criteria, so-called Sapporo criteria, that defined patients with APS were set in year 1998, with a revision in 2006. The Sapporo classification divided the APS criteria into clinical and laboratory. APS is present if at least one of the clinical criteria and one of the laboratory criteria are met. Diagnosis of APS should be avoided if less than 12 weeks or more than 5 years separate the positive aPL test and the clinical manifestation^2^.

**List.** The Sapporo classification criteria for APS

APS is present if at least one clinical criteria and laboratory criteria are met.

### - CLINICAL CRITERIA

#### 1. *Vascular thrombosis* †

1.2. One or more clinical episodes‡ of arterial, venous, or small vessel thrombosis§, in any tissue or organ. Thrombosis must be confirmed by objective validated criteria (appropriate imaging studies). For histopathologic confirmation, thrombosis should be present without significant evidence of inflammation in the vessel wall.

#### 2. Pregnancy morbidity

2.1. One or more unexplained deaths of a morphologically normal fetus at/or beyond the 10th week of gestation, with normal fetal morphology documented by ultrasound or by direct examination of the fetus, or

2.2. One or more premature births of a morphologically normal neonate before the 34th week of gestation because of: (i) eclampsia or severe pre-eclampsia defined according to standard definitions, or (ii) recognized features of placental insufficiency¶, or

2.3. Three or more unexplained consecutive spontaneous abortions before the 10th week of gestation, with maternal anatomic or hormonal abnormalities and paternal and maternal chromosomal causes excluded.

### - LABORATORY CRITERIA

1. Lupus anticoagulant (LA) present in plasma, on two or more occasions at least 12 weeks apart, detected according to the guidelines of the International Society on Thrombosis and Haemostasis^5^.

2. Anticardiolipin (aCL) antibody of IgG and/or IgM isotype in serum or plasma, present in medium or high titer, on two or more occasions, at least 12 weeks apart, measured by a standardized ELISA^6^.

3. Anti-β2 glycoprotein‐I antibody of IgG and/or IgM isotype in serum or plasma, present on two or more occasions, at least 12 weeks apart, measured by a standardized ELISA^7^.

## CASE PRESENTATION

In January 2018, a 32-year-old female patient was admitted to the hospital because of malfunction of the temporary vascular access for hemodialysis. Her medical history presented miscarriage, pre-eclampsia with preterm delivery, chronic kidney disease (CKD) stage 5 on renal replacement therapy, and recurrent thrombosis of arteriovenous fistulas (AVFs).

She had a miscarriage at 7^th^ week of gestation in October 2015. CKD stage 4 was diagnosed immediately after the miscarriage. The patient presented high blood pressure, elevated serum level of creatinine (190 µmol/L), and urinary protein level of 3 grams per diuresis. The renal biopsy demonstrated global and segmental glomerulosclerosis, tubular atrophy with thyroidization, and arteriosclerosis. Immunopathologic assessment of the renal tissue showed C3 linear deposition along the glomerular basement membrane and mesangium.

The patient’s second pregnancy was complicated by impaired kidney function and pre-eclampsia. The preterm delivery was performed at 28^th^ week of gestation in July 2017. Her second pregnancy finished successfully with live birth of a premature baby girl with weight of 890 grams and APGAR score 5/6. Some complications of prematurity were registered in the new-born during the early and late neonatal period. The hemostasis testing showed shortened activated partial thromboplastin time (aPTT) of less than 23 s with an elevated D-dimer (4102 ng/mL). The patient started with hemodialysis treatment immediately after the preterm delivery.

Her maintenance hemodialysis care was complicated by recurrent vascular access thrombosis. The patient had multiple AVFs that failed due to thrombosis. The first distal (radial-cephalic) AVF and second proximal (brachial-cephalic) AVF were created on the left forearm. A computer tomography angiogram was performed on the patient’s right arm before the planned creation of AVF on right arm. The angiogram showed blood vessels without deformation (stenosis or thrombosis). Repeated hemostasis testing showed shortened aPTT of less than 25 s with an elevated D-dimer (1017 ng/mL). Therapy with low-molecular-weight heparin (Enoxaparin, 40 mg/0.4 mL) with dosage of 0.7 mg/kg was started one week before the intervention. The middle (radial-cephalic) AVF was created on the patient’s right forearm. The third AVF also did not function due to thrombosis. Central venous catheters were necessary for a proper vascular access, with all the difficulties and hazardous situations.

A thorough thrombophilia screening confirmed the presence of aPL. LA was present in plasma, measured on two occasions 12 weeks apart. Anticardiolipin antibody (IgG and IgM) and anti-*β*_2_ glycoprotein-I antibody (IgG and IgM) were within normal range. Antiphosphatidylserine antibodies (APSA) were also negative. To rule out an APS secondary to systemic lupus erythematosus, the patient was evaluated and found negative for antinuclear and anti-dsDNA antibodies on several occasions. Treatment with long-term anticoagulant therapy with a vitamin K antagonist was started with target international normalized ratio (INR) 2 to 3. The peritoneal dialysis was planned as a renal replacement therapy for this patient with recurrent thrombosis of AVFs.

## DISCUSSION

This case report described a patient with primary APS characterized by recurrent vascular access thrombosis, obstetrical complications, and positive LA.

Obstetrical complications were present in the medical history with one miscarriage at 7^th^ week of gestation and development of pre-eclampsia with preterm delivery at 28^th^ week of gestation. The aPL antibodies can bind to phospholipids in the cell membranes initiating an endothelial cell activation and defective placentation or activation of the complement system triggering a cascade of reactions, which produce pro-inflammatory substances that damage the products of conception, either the placenta or the fetus itself^8^.

The patient also developed APS-associated nephropathy (APSN), which progressed to CKD stage 5 with the necessity of renal replacement therapy. Clinically, hypertension is one of the first major features of APSN. In addition, patients frequently have CKD with acute and/or mild levels of proteinuria that can progress to nephrotic-range proteinuria. The glomerular lesions may be acute (thrombotic microangiopathy) and/or chronic (arteriosclerosis, arterial fibrous intimal hyperplasia, tubular thyroidization, arteriolar occlusions, and focal cortical atrophy)^9^. There are also glomerulopathies associated with APS such as membranous nephropathy, minimal change disease, focal segmental glomerulosclerosis, mesangial C3 nephropathy, and pauci-immune crescentic glomerulonephritis^10^.

The maintenance of adequate vascular access is crucial to patient survival on hemodialysis. Options for vascular access include catheter, arteriovenous graft (AVG) and AVF. Thrombosis is the leading cause of failure of the AVF. It is also the leading cause of permanent vascular access loss. Access thrombosis accounts for 65 to 85% of cases of permanent vascular access loss^11^. In the present case, the hemodialysis treatment was complicated by recurrent thrombosis of the AVFs. Virchow’s triad of endothelial injury (endothelial dysfunction, stasis (stenosis), and hypercoagulability (thrombophilia)) could be applied to vascular access thrombosis^12^. Thrombophilia in an inherited or acquired predisposition to thrombosis and has been suggested as a possible cause of vascular access thrombosis. Thrombophilic disorders have been associated with both arterial and venous thrombosis. A case-control study was conducted on 419 patients on hemodialysis to determine whether thrombophilia was associated with AVF or graft thrombosis^13^. Patients were tested for factor V Leiden, prothrombin gene mutation, factor XIII genotype, methylenetetrahydrofolate reductase genotype, LA, anticardiolipin antibody, factor VIII, homocysteine, and lipoprotein(a) concentrations. The study suggested that thrombophilia was associated with vascular access thrombosis in patients on hemodialysis (adjusted OR, 2.42; 95% CI, 1.47 to 3.99, *p* = 0.001).

The case report presented a patient on hemodialysis with primary APS as a cause for the recurrent thrombosis of the AVFs. However, several studies pointed out that an elevated aPL levels have been found in patients with CKD stage 5 when compared to the general population. Moreover, the prevalence of aPL was higher among patients treated by hemodialysis than in CKD stage 5 patients on conservative treatment or on peritoneal dialysis. The mechanism of aPL increase in these patients is unknown. There was a suggestion that hemodialysis membrane biocompatibility plays a role in the occurrence of aPL antibodies^14^. Certain aPLs, in particular LA, was more strongly associated with the thrombosis than other aPLs, especially more commonly associated with venous thrombosis than with arterial thrombosis^15^. Vascular access thrombosis were significantly more frequent in patients on hemodialysis with LA than in those without LA (62 vs. 26%; *p* = 0.010). The highest prevalence of aPL was found in patients on hemodialysis with unknown etiology of renal disease. Although the preventive treatment of vascular access thrombosis in patients on hemodialysis is controversial, patients with a history of thrombosis and presence of LA should be evaluated about the need for anticoagulant therapy with a vitamin K antagonist^16^.

The treatment of patients with APS defined by venous thrombosis starts with low-molecular-weight heparin, followed by long-term anticoagulant therapy with a vitamin K antagonist (INR 2 to 3). Recurrent venous thrombosis despite long-term anticoagulant therapy is a well-recognized complication of APS. When therapy with vitamin K antagonist fails despite a therapeutic INR, options include high-intensity dosage with target INR 3 to 4; the addition of low-dose aspirin, hydroxychloroquine, or a statin; use of a different anticoagulant, such as low-molecular-weight heparin; and a combination of these approaches^17^. Direct oral anticoagulants were less effective than a vitamin K antagonist in the prevention of recurrent thrombosis in patients with a high-risk profile (those who are triple-positive for LA, anticardiolipin antibodies, and anti-β_2_-glycoprotein I antibodies)^18^.

## Footnotes

† Coexisting inherited or acquired factors for thrombosis are not reasons for excluding patients from APS differential diagnosis. However, two subgroups of APS patients should be recognized, according to: (a) the presence, and (b) the absence of additional risk factors for thrombosis. Indicative such cases include: age (> 55 in men, and > 65 in women), and the presence of any of the established risk factors for cardiovascular disease (hypertension, diabetes mellitus, elevated cholesterol, cigarette smoking, family history of premature cardiovascular disease, body mass index ≥ 30 kg/m^2^, microalbuminuria, estimated glomerular filtration rate < 60 mL/min), inherited thrombophilias, oral contraceptives, nephrotic syndrome, malignancy, immobilization, and surgery. Thus, patients who fulfill criteria should be stratified according to contributing causes of thrombosis.

‡ A thrombotic episode in the past could be considered as a clinical criterion, provided that thrombosis is confirmed by appropriate diagnostic means and that no alternative diagnosis or cause of thrombosis is found.

§ Superficial venous thrombosis is not included in the clinical criteria.

¶ Generally accepted features of placental insufficiency include: (i) abnormal or non-reassuring fetal surveillance test, e.g. a non‐reactive non‐stress test, suggestive of fetal hypoxemia, (ii) abnormal Doppler flow velocimetry waveform analysis suggestive of fetal hypoxemia, e.g. absent end-diastolic flow in the umbilical artery, (iii) oligohydramnios, e.g. an amniotic fluid index of 5 cm or less, or (iv) a post-natal birth weight less than the 10th percentile for the gestational age.
